# Clinical Application of Mesenchymal Stem Cells and Novel Supportive Therapies for Oral Bone Regeneration

**DOI:** 10.1155/2015/341327

**Published:** 2015-05-12

**Authors:** Miguel Padial-Molina, Francisco O'Valle, Alejandro Lanis, Francisco Mesa, David M. Dohan Ehrenfest, Hom-Lay Wang, Pablo Galindo-Moreno

**Affiliations:** ^1^Department of Periodontics and Oral Medicine, School of Dentistry, University of Michigan, Ann Arbor, MI 48109, USA; ^2^Department of Oral Surgery and Implant Dentistry, School of Dentistry, University of Granada, 18009 Granada, Spain; ^3^Research Group #CTS-583 (Implantology and Periodontics), University of Granada, Junta de Andalucía, Spain; ^4^Department of Pathology, School of Medicine, University of Granada, 18009 Granada, Spain; ^5^Biopathology and Regenerative Medicine Institute (IBIMER), University of Granada, 18009 Granada, Spain; ^6^Research Group #CTS-138 (Pathology), University of Granada, Junta de Andalucía, Spain; ^7^Private Practice, 8320000 Santiago, Chile; ^8^Implantology Program, University of San Sebastian, 8320000 Santiago, Chile; ^9^Department of Periodontics, School of Dentistry, University of Granada, 18009 Granada, Spain

## Abstract

Bone regeneration is often needed prior to dental implant treatment due to the lack of adequate quantity and quality of the bone after infectious diseases, trauma, tumor, or congenital conditions. In these situations, cell transplantation technologies may help to overcome the limitations of autografts, xenografts, allografts, and alloplastic materials. A database search was conducted to include human clinical trials (randomized or controlled) and case reports/series describing the clinical use of mesenchymal stem cells (MSCs) in the oral cavity for bone regeneration only specifically excluding periodontal regeneration. Additionally, novel advances in related technologies are also described. 190 records were identified. 51 articles were selected for full-text assessment, and only 28 met the inclusion criteria: 9 case series, 10 case reports, and 9 randomized controlled clinical trials. Collectively, they evaluate the use of MSCs in a total of 290 patients in 342 interventions. The current published literature is very diverse in methodology and measurement of outcomes. Moreover, the clinical significance is limited. Therefore, the use of these techniques should be further studied in more challenging clinical scenarios with well-designed and standardized RCTs, potentially in combination with new scaffolding techniques and bioactive molecules to improve the final outcomes.

## 1. Introduction

Hard and soft tissues in the oral cavity are constantly being challenged. As a consequence of infectious oral diseases, trauma, tumor or cyst resection, or congenital and developmental conditions (i.e., cleft palate defects), tooth loss results in the alteration of basic functional, aesthetical, and psychological needs. Mastication, speech, swallowing, and thermal and physical protection of important anatomical structures (i.e., brain, nerves, arteries, and veins) are diminished [1]. In these situations, tooth replacement by osseointegrated implants is an essential tool to restore the normal stomatognathic system. However, quantitative and qualitative proper bone architecture to allow successful implant treatment is, unfortunately, sometimes compromised. If the adequate bone is not restored previously to the implant treatment, it may lead to further complications [2, 3].

Bone deficiencies in the oral cavity differ enormously in extension and etiology, ranging from localized alveolar bone loss due to periodontal disease to extensive bone atrophy as a consequence of a variety of syndromes, including traumatic injuries and bone resorption associated with a number of benign or malignant tumors. Extensive bone deficiencies, in particular, are really challenging in the clinical setting [4].

Bone regeneration requires the migration of specific cells to the healing area to proliferate and provide the biological substrate for the new tissue to grow. Soluble factors, different cell types, extracellular matrix (ECM), and matricellular proteins mediate and coordinate this process. Initially, angiogenic signals and new vascular networks provide the nutritional base for tissue growth and homeostasis. Simultaneously, a three-dimensional template structure based on a proper extracellular matrix is synthesized and organized. This template will, later, support and facilitate the process of bone formation and maturation. Once those structures are established, the regenerated bone will go on under the normal homeostatic and modeling-remodeling processes [5, 6].

**Figure 1:**
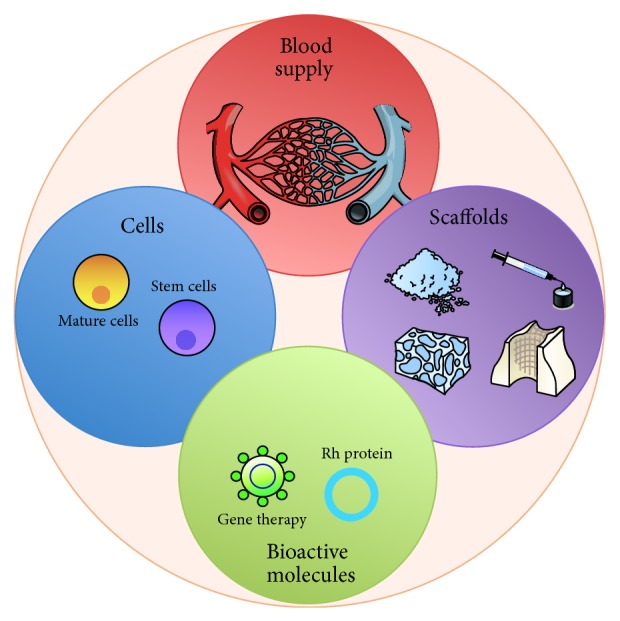
Schematic requirements for bone regeneration from a tissue engineering perspective.

Although the exact mechanisms that regulate the bone regeneration process at the deepest biomolecular level are yet to be understood, several methods for predictable bone reconstruction have been proposed [7], ranging from autografts, to allografts, xenografts, and alloplasts. These techniques present different drawbacks including the limited availability of autografts and their associated morbidity in addition to the absence of cell populations carried by allografts, xenografts, and alloplasts, which determine poor osteoinductive properties. To overcome these limitations, the use of growth factors incorporated in carriers, the stimulation of the selective production of growth factors using gene therapy, and the delivery of expanded cellular constructs are being used in different areas of maxillofacial reconstruction [8] (Figure 1). Cell therapy approaches constitute one of the most promising instruments to enhance reconstruction of both hard and soft tissues.

Stem cells are unspecialized cells with the ability to proliferate and differentiate to multiple cell types when stimulated by both internal and external signals. Adult (somatic) stem cells that exhibit this plasticity are called pluripotent cells and can be found in bone marrow in the form of hematopoietic, endothelial, and mesenchymal (stromal) stem cells (MSCs). Other sources of MSCs in adult patients have been also identified such as adipose tissues (ASCs), lung, and teeth (perivascular niche of dental pulp and periodontal ligament) [9, 10] (Table 1). Mesenchymal and Tissue Stem Cell Committee of the International Society for Cellular Therapy proposes minimal criteria to define human MSC. Firstly, MSC must be plastic-adherent when maintained in standard culture conditions. Secondly, MSC must express CD105, CD73, and CD90 and lack expression of CD45, CD34, CD14 or CD11b, CD79a, or CD19 and HLA-DR surface molecules. Thirdly, MSC must differentiate to osteoblasts, adipocytes, and chondroblasts in vitro [11]. In this way, MSCs can produce bone, cartilage, fat, or fibrous connective tissue depending on their differentiation process [10] and, therefore, are of most interest in the area of dental implantology. Different technologies and application protocols are being studied in this area. However, it is still needed to identify the appropriate cell types, origin, and processing protocols as the most critical determinants to achieve successful outcomes [12]. Due to the limited availability of MSCs from bone marrow, ASCs are also being explored. Adipose tissue is a rich source for multipotent stromal/stem cells (adipose-derived mesenchymal stromal/stem cells or ASCs) and has several advantages compared to other sources of mesenchymal stromal/stem cells (ubiquitous available, easy accessible source by liposuction, and more abundant 0.5–2 × 10^6^ AScs/g adipose tissue) [13, 14].

Therefore, the main purpose of this review is to identify the existing literature on clinical studies utilizing MSCs or ASCs to treat oral bone defects and to critically analyze their validity, methodology, and outcomes. Additionally, emerging strategies for the recruitment and transplantation of MSCs into bone defects will also be discussed.

## 2. MSC-Based Bone Regeneration

### 2.1. Materials and Methods

A search of electronic databases including Ovid (MEDLINE), PubMed, and Cochrane Central for studies was performed in September 2014 by two examiners limited to articles published in English during the last 10 years performed on human subjects. The search build used was as follows: (“Mesenchymal Stem Cell Transplantation” [Mesh] OR “Adult Stem Cells” [Mesh] OR “Stem Cells” [Mesh] OR “Stem Cells Transplantation” [Mesh] OR “Tissue Therapy” [Mesh] OR “Bone Marrow Transplantation” [Mesh] OR “Bone Marrow” [All Fields] OR “stem cell therapy” [All Fields] OR “stem cell” [All Fields]) AND (“Sinus Floor Augmentation” [Mesh] OR “Bone Regeneration” [Mesh] OR “Alveolar Ridge Augmentation” [Mesh] OR “craniofacial bone regeneration” [All Fields] OR “craniofacial” [All Fields] OR “alveolar bone” [All Fields] OR “implant site development” [All Fields]) AND ((Controlled Clinical Trial [ptyp] OR Clinical Trial [ptyp] OR Randomized Controlled Trial [ptyp] OR Case Reports [ptyp] OR Comparative Study [ptyp] OR Validation Studies [ptyp] OR Evaluation Studies [ptyp]) AND “2004/09/12” [PDat]: “2014/09/12” [PDat] AND “humans” [MeSH Terms] AND English [lang]).

In addition, a manual search was conducted in related scientific journals and relevant papers that could contribute to the process of information collecting.

The following inclusion criteria to select the articles obtained after the search were as follows: human clinical trial (randomized or controlled) and case reports/series on the clinical application of MSCs in oral bone regeneration. On the other hand, articles were excluded if the technique applied was related to periodontal regeneration or was not associated with bone tissue reconstruction. Articles were first screened by analyzing the abstract. From those which were selected in this phase, full-text was obtained and analyzed for a second screening. Potential articles were independently reviewed in full-text by two examiners. The final decision on the included articles was made with mutual agreement of the two examiners.

Additionally, a critical review of relevant supportive technologies for bone regeneration in combination with MSCs has been conducted.

### 2.2. Results

**Figure 2:**
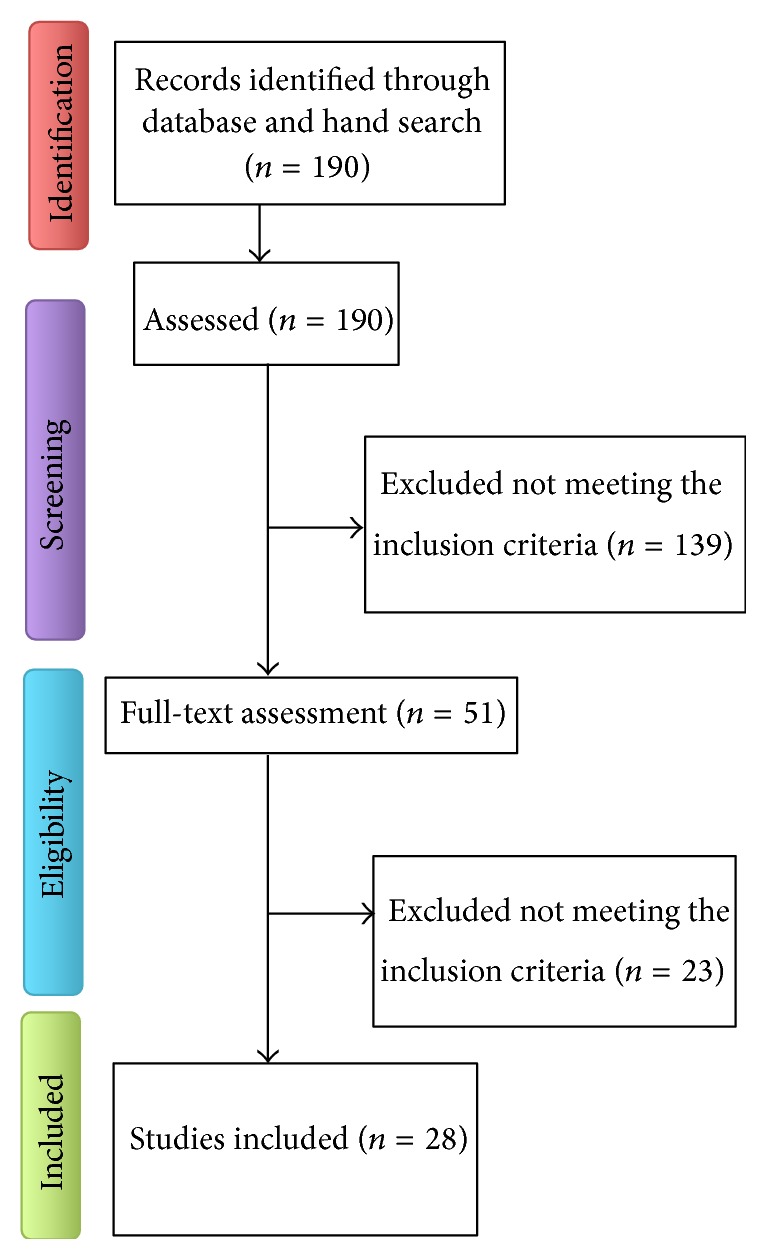
Flow chart of the paper selection process.

A total of 190 records were identified by the database and hand search and were assessed for eligibility. After reading the abstracts, 51 articles were selected for full-text assessment. Of those, only 28 were included in this review based on the inclusion criteria previously determined. From the 28 articles selected (Figure 2), 9 corresponded to randomized controlled clinical trials [15–23] (Table 2), 9 to case series [24–26, 32, 36–40], and 10 to case reports [27–31, 33–35, 41, 42] (Table 3). Collectively, they evaluate the use of MSCs in a total of 290 patients/342 interventions. However, due to the high variability among different variables, a meta-analysis was not considered appropriate.

Bone deficiencies in the oral cavity differ enormously in extension and etiology, ranging from localized alveolar bone loss due to periodontal disease to extensive bone atrophy as a consequence of a variety of syndromes, including traumatic injuries and bone resorption associated with a number of benign or malignant tumors. In these clinical scenarios, functional and esthetical rehabilitation by dental implants is an essential tool. However, a proper quantity and quality of bone is a prerequisite not always present [1]. Therefore, different regenerative techniques have been proposed in these scenarios aiming at achieving predictable outcomes. Extensive bone deficiencies, in particular, are really challenging in the clinical setting. Fortunately, cell transplantation strategies can provide a viable treatment option to overcome the limitations of autograft harvesting and the reduced colonization of nonautograft materials and constitute one of the most promising instruments to enhance reconstruction of both hard and soft tissues [43, 44].

Bone regeneration requires not only osteolineage populations to migrate, proliferate, and differentiate into the treated area but also, of extreme importance, angiogenesis to provide the adequate nutrients and environment in which the bone tissue can grow and develop [12, 45]. Because of this, stem cells have gained interest due to their capacities to differentiate to a variety of cell lineages, including hematopoietic, mesenchymal, and endothelial cells [10]. Stem cells can be found in different tissues, such as bone marrow, adipose tissue, and, in the oral cavity, periodontal tissue, dental pulp, and dental follicle [8] (Table 1). However, due to the limited autogenous availability in some of those locations, only bone marrow and adipose-derived mesenchymal stromal/stem cells have been clinically applied to bone regeneration in the oral cavity. Although different technologies and application protocols are being studied in this area, the optimal cell type, origin, and processing protocol are yet to be identified [12].

The analysis of the published literature on the clinical use of MSCs for oral bone regeneration previous to dental implant placement highlights the lack of proper RCTs with comparable methodologies to extract proper overall conclusions. However, out of the 28 identified clinical studies, 25 report the use of iliac bone marrow aspirates (BMA) which reflects that this location is widely accepted as the current standard for aspirate harvesting [16–30, 33–42]. In fact, BMA from the iliac crest has been confirmed as the harvesting technique with less morbidity and better patient comfort than the traditional bone harvesting from the same location [19].

However, there is no standardization in terms of the processing and handling of such aspirates. While some studies use an expansion and isolation protocol previous to the surgical implantation (with a variety of subculture times, culture supplementations, automated or manual processes, cell population selection, etc.), others do the aspirate intrasurgically (chairside) and use the whole aspirate or a commercially available concentration kit (to select endothelial progenitors, hematopoietic and mesenchymal stem cells, platelets, lymphocytes, and granulocytes) (BMAC Harvest Technologies Corporation, Plymouth, MA, USA). One RCT compared the use of nonprocessed BMA versus PRP or CD34+ cells (angiogenic cells isolated from a BMA). Radiographic results from this study confirmed the utility of BMA and CD34+ over PRP alone [23]. However, to our knowledge, no clinical comparison has been done between processed BMA and nonprocessed BMA, which will be of high interest. In this sense, results from 2 RCTs [20, 21] show a similar achievement in postextraction socket reconstruction in terms of horizontal and vertical augmentation, even though the cell concentration procedure was performed by an automatic cell culture system (that specifically increases the proportion of bone repair cells, that is, hematopoietic and mesenchymal stem cells) (Ixmyelocel-T, Aastrom Biosciences Ann Arbor, MI, USA) [20] or a chairside technique including the whole aspirate with no concentration step [21].

Another important difference amongst studies is the carrier used to deliver the cells. It ranges from alloplastic graft (*β*-TCP or HA) to xenograft (mainly bovine bone), allograft or autograft (either PRP concentrate or autogenous bone). Other studies use a combination of those materials, with or without the addition of additional factors such as PDGF or BMP-2. No standardization is found on the use of a covered membrane over the grafted area either.

Additionally, different defects are being treated in these studies. Those defects range from extensive non-self-contained (cleft palate and tumoral postresection defects) to extensive self-contained (sinus lift), nonextensive self-contained (postextraction sockets), and nonextensive non-self-contained defects (vertical and horizontal alveolar ridge augmentation). Bone regeneration in these situations differs enormously from one to another.

**Figure 3:**
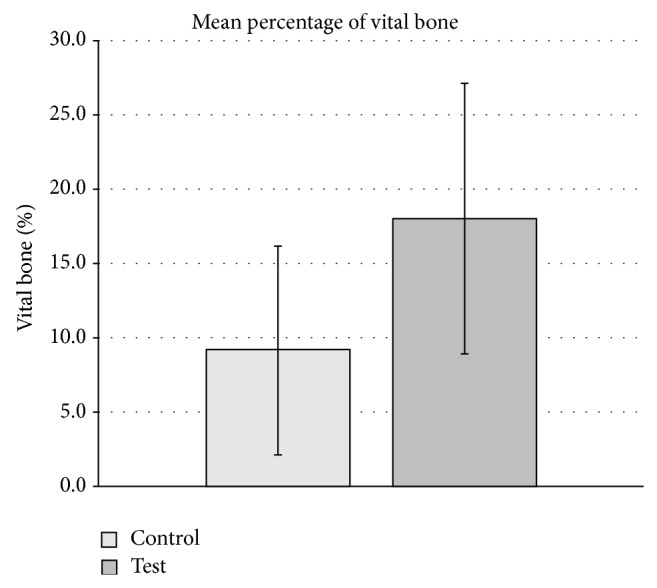
Weighted mean percentage of vital bone from RCTs on sinus lift [15–17]. No overall statistical significance difference was found (*p* = 0.085, Student's *t*-test).

Globally, the results in most of the available literature show the goodness of the technique by vague subjective indications of qualitative appreciations and some studies fail to report specific objective quantitative data. When they do, the reported data is not comparable either as it ranges from vertical, to horizontal, or volumetric measures. Additionally, these measures are presented in absolute magnitudes or % of gain or reduction depending on the study. On the other hand, the number of differences among the identified RCTs makes it difficult to make a fair global comparison. Only 2 of those RCTs are fairly comparable as they use similar methodologies for concentration process (chairside), cell origin (iliac crest), defect type (sinus lift), and control group (bovine bone + autogenous graft) [16, 17]. From both studies that globally treated 69 sinuses (46 tests and 23 controls) in 38 patients, it can be concluded that the combination of bovine bone plus BMA concentrated chairside provides a higher radiographic volume gain of 1.74 ± 0.69 versus 1.33 ± 0.62 mL (test versus control, *p* < 0.02) [17] and better histological outcomes in terms of new bone formation 17.7 ± 7.3 versus 12.0 ± 6.6% (test versus control, *p* < 0.026) [16]. Sauerbier et al. [17] reported no histological differences (12.6 ± 1.7 versus 14.3 ± 1.8%; test versus control, *p* = 0.333). Other studies on sinus lift also show histological advantage of using stem cells carried in an allograft cellular bone matrix in this location (new vital bone: 32.5 ± 6.8% versus 18.3 ± 10.6%, test versus control) [15]. The weighted mean percentage of vital bone obtained by these studies is not statically different from control (9.14 ± 7.02) to test group (18.02 ± 9.1) (*p* = 0.085, Student's *t*-test) (Figure 3). The differences are even more diffuse if other studies treating other defect locations are included in this comparison. This highlights the necessity for better-designed studies to reduce bias and variety of data and ultimately enables consensus in this field.

In summary, the main overall report findings were that the clinical application of stem cells for oral bone regeneration promotes better outcomes in terms of clinical, radiographic, and histological parameters. However, the clinical significance in the applications analyzed in those RCTs (mainly self-contained defects, that is, postextraction sockets and sinus floor elevation) is very limited. Therefore, it could be argued that (1) the use of stem cells is not necessary in small defects that can be successfully treated by other means and (2) the lack of conclusive advantages does not surpass the scientific doubts, morbidity, and potential complications that stem cell therapy may possess. Therefore, the generalizability for the use of stem cell therapy in the daily clinical setting is still to be confirmed and probably not recommended for many clinical cases. Its advantages are yet to be studied in more challenging scenarios, such as extensive non-self-contained defects (vertical alveolar bone augmentation, extensive bone deficiencies in postresection tumor defects, and cleft palate conditions) where they may show their greatest potential over current treatment options.

## 3. Novel Supportive Strategies for the Use of MSCs in Bone Regeneration

The clinical use of MSCs for oral bone regeneration is usually accompanied by supporting scaffolds and bioactive molecules to further increase the capabilities of cell-based therapies.

### 3.1. Scaffolds for Bone Regeneration

The main purpose of a scaffold is to provide a mechanical support for cell migration, proliferation, and activity by mimicking the ECM. They will stimulate the production and maturation of a new ECM that will eventually mineralize. A scaffold will ideally provide a template for the subsequent bone formation, which starts in the periphery and continues towards the inner part. In this process, porosity is of extreme importance since it will facilitate cell ingrowth and vascularization and the biodegradation process [46]. Additionally, surface chemistry, surface charge, and topography are also important in the interactions between cells and material for bone tissue growth [5, 47].

Conventional scaffolds naturally derived (autografts, allografts, and xenografts) or synthetic materials (alloplasts) are commonly used in bone regeneration and implant therapy [5]. However, clinical needs of full control of the regeneration process, focus, and orientation and in large defects cannot be successfully achieved by this classic or conventional approach. Fortunately, additive manufacturing processes allow full control of porosity features, 3D structure, and surface properties of the synthesized material and it is, therefore, being the focus of extensive research [48]. They can be combined with cell-, growth factor- or gene-based approaches to serve as supportive carriers and induce stimuli for tissue formation [49]. 3D printed scaffolds can also mimic stem cell niches to regulate daughter cell proliferation, differentiation, and dispersion into the surrounding tissue or by attracting useful cells to a desired anatomic site [49, 50].

Additive manufacturing is defined as the process of joining materials to make objects from 3D model data, usually layer upon layer, as opposed to subtractive manufacturing methodologies [51]. Usually, 3D printing is used as a synonym since it is the most widely used. However, additive manufacturing also includes other scaffold fabrication techniques such as solid freeform fabrication (SFF) and rapid prototyping (RP) that use deformation and solidification, polymerization, laser-assisted sintering, or direct writing-based processes to create the final scaffold [52]. Additive manufacturing relies upon computer-based scaffold design and fabrication [53]. This image-based design technology can be used to define virtual three-dimensional models of anatomic geometry of the defect and to create a template for the scaffold on a global anatomic level [54]. By additive manufacturing, the heterogeneous structures to be regenerated can be mimicked by variations in macro-, micro-, and nanostructures and scaffold surface topography, which will influence the modulus of elasticity, permeability, and cell orientation [55–57].

For bone regeneration, a large variety of ceramic, polymeric, and composite materials can be processed using 3D printing to control interconnected porosity [52]. Among these, calcium phosphates are the main materials used in bone engineering. Of them, hydroxyapatite (HA) is the most used and studied ceramic material in the dental field [8]. HA possesses an excellent bioactivity, osteoconductivity and is similar to bone in composition. It can stimulate BMP-2 expression in a pathway dependent on the p38 MAP kinase [58], increase of capillaries and vessel formation, and homogeneous osteoconduction from central channels with no cytotoxicity and adequate cell adhesion [59]. Hydroxyapatite customized scaffolds can be combined with MSCs to achieve better results than those obtained with controls [60]. Similarly, *β*-tricalcium phosphate, a synthetic inorganic calcium-phosphate-based material, has demonstrated an increase of human osteoblasts ingrowth, proliferation, and new bone formation [61] with adequate biocompatibility also confirmed by tartrate resistant acid phosphatase (TRAP) staining and lacunae formation [62].

Synthetic polymers in bone tissue engineering are very flexible [63]. Property modification, control of macrostructure, degradation time and release mechanism, and exposure duration of bioactive molecules can be better controlled with these materials [64–66]. It is possible to maintain the therapeutic levels of encoded proteins and to limit unwanted immune response and potential side effects. The most studied synthetic materials for additive manufacturing are the group of poly(lactic-co-glycolic acid)based (PLGA) biomaterials. This material has been successfully used for the regeneration of complex bone-ligament interfaces with rapid prototyping techniques [54, 55, 67]. Other polymers under study are the group of poly(ethylene glycols) and poly(*ε*−caprolactone) methyl ethers. They can be combined with cell transplantation and hydroxyapatite to increase the mimetic properties and, therefore, improve bone regeneration results, specifically in terms of mechanical strength [68].

Based on these properties, the use of 3D printing in fabricating scaffolds with live osteoprogenitor cells [69] and the controlled delivery of specific growth factors such as BMP-2 [59, 70], collagen, and chondroitin sulfate [71] or other drugs like tetracycline [72] are being explored. The potential application is to reduce the dose of those molecules, control the release pattern, and reduce side effects. Bioprinting, however, is still at a very early stage and much research is yet to be done.

### 3.2. Bioactive Molecules and Gene-Therapy Techniques

The delivery of growth factors and other bioactive molecules was the first approach into using a biological agent modifier for regeneration purposes [73]. A number of factors, such as platelet derived growth factor (PDGF), fibroblast growth factor (FGF), insulin growth factor (IGF), bone morphogenic proteins (BMPs, specially BMP-2 and BMP-7), periostin, vascular endothelial growth factor (VEGF), and transforming growth factor beta (TGF-*β*), are present in the healthy bone matrix and are expressed during bone healing [74]. They regulate vascularization and induce proliferation and differentiation of osteoprogenitors cells [52, 61, 70] and surrounding tissues in the periodontal and gingival structures [75–78]. Therefore, they can be useful for improving the healing processes and to stimulate bone regeneration and have extensively studied and reviewed elsewhere [8, 49].

**Figure 4:**
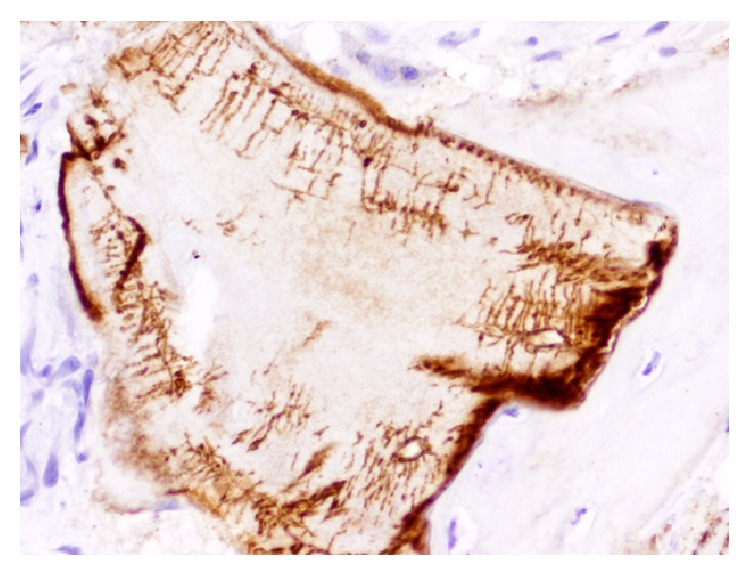
Osteopontin immunohistochemical detection on anorganic bovine bone particle (Bio-Oss). Note bone formation where intense interstitial expression of OPN is observed in a case of maxillary sinus floor elevation (micropolymer peroxidase-based method, original magnification ×20).

In addition to the mentioned growth and/or transcription factors and regulators of osteogenesis, our group has recently initiated efforts in investigating the potential of two molecules that can be of interest in this topic as well. Osteopontin (OPN) is a highly phosphorylated sialoprotein abundant in the mineralized extracellular matrices of bones and teeth [79]. OPN, mainly through its RGD region, stimulates cell activity to influence, amongst others, bone formation, remodeling, and maintenance as well as angiogenesis [80]. In bone, it is primarily synthesized by cells of osteoblastic lineage and in wound healing sites [81] where it interacts with the cell-surface receptor CD44 [82]. This interaction has been shown to increase MSCs recruitment to the bone healing area [83]. Previous findings related OPN expression to the presence of CD44-positive cells in anorganic bovine bone (ABB) particles in samples from sinus floor augmentation. OPN was found on the interstitial boundary of new bone with ABB, inside lacunae spaces, bone canaliculi and in osteocytes in trabecular bone without expression in the trabecular bone or the interstitium [84, 85]. Therefore, combining scaffold modification with the delivery of bioactive molecules, it could be possible to include OPN in ABB particles to increase bone regeneration in vivo (Figure 4).

**Figure 5:**
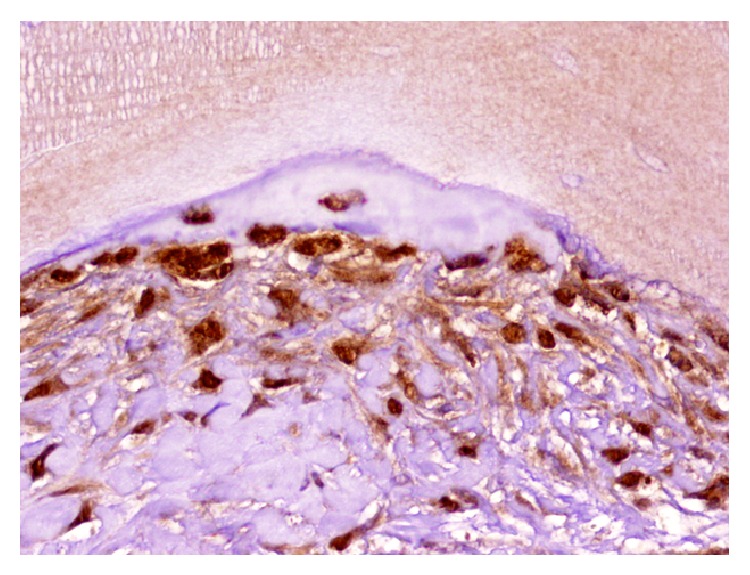
Immunohistochemical expression of Musashi-1 in fusocellular cells, osteoblasts, and osteocytes in a case of maxillary sinus floor elevation with anorganic bovine bone (micropolymer peroxidase-based method, original magnification ×20).

Another important attractor of MSCs to the bone healing area is Musashi-1. Musashi-1 is an osteogenic marker expressed in osteoblasts (cytoplasm and nuclei) and osteocytes (nuclei) (Figure 5). It binds to RNA as a translational regulator in MSCs with osteogenic capacities. It could therefore be delivered to increase recruitment and differentiation of osteogenic MSCs [86]. These activities are still at their very infancy and more research is needed.

However, an important drawback of these described methods is the difficulty in activating the right process at the right location in the right cells at the right time for a sufficient amount of time, while minimizing adverse reactions [87]. Important advances have been made to overcome these limitations. Some of the most promising strategies are those designed to activate the protein release from the scaffold [88] or the activation of the bioactive molecule [89] “on demand” by an external source or trigger. Different methodologies are under study, including the activation by changes in pH, proteases activity, and energy-based stimuli such as magnetism, electricity, light, and temperature [88]. Focused ultrasound is an emerging clinical technology primarily used for the thermal and/or mechanical ablation of cancerous or precancerous tissues deep within the body that can be focused on small volumes and generate spatially restricted regions of hyperthermia by coupling a magnetic resonance imaging (MRI) instrument to an integrated high intensity focused ultrasound (HIFU) transducer. HIFU has also been successfully used to achieve spatial and temporal control of the production of VEGF and BMP-2 in vitro and in vivo [89, 90]. With such technology, scaffolds, cells, bioactive molecules, and gene therapies could be suitable for a 4D control. The implications of this approach range from the identification of spatiotemporal patterns of gene expression during development to, more importantly within the aim of this review, the application of those patterns for bone regeneration in vivo as a future clinical tool.

### 3.3. Perspectives on Platelets and Fibrin Gels for Combined Therapies

In last decades, many different approaches have been attempted for the use of autologous platelet concentrates that serve as both carrier and metabolic stimulators through their high concentration of growth factors [91]. Despite the specific differences between them in terms of concentration procedure, coagulant treatment, separation of blood phases, and so forth [92], they have been tested in many different clinical fields, such as oral and maxillofacial surgery, ear-nose-throat surgery, plastic surgery, orthopaedic surgery, sports medicine, gynecologic and cardiovascular surgery, and ophthalmology [91, 93]. In the literature, their clinical and experimental results are often controversial and difficult to sort and interpret [94, 95], mostly because of the lack of proper terminology between many different families of products, often wrongly regrouped under the inaccurate generic term PRP (platelet-rich plasma) [92]. Recent terminology and classification regrouped these techniques into 4 families depending on their fibrin architecture and cell content [92, 96, 97]: pure platelet-rich plasma (P-PRP), leukocyte- and platelet-rich plasma (L-PRP), pure platelet-rich fibrin (P-PRF), and leukocyte- and platelet-rich fibrin (L-PRF).

The 2 families of PRPs are first of all platelet suspensions, which can jellify after activation like a fibrin glue [96, 98]; as it was discussed previously, the PRP fibrin gels were tested as scaffolds for MSCs, as fibrin gels are quite common scaffolds in tissue engineering [91]. On the other hand, the 2 families of PRF only exist in a strongly polymerized fibrin gel form [99]. P-PRP and P-PRF do not contain leukocytes or any other cell bodies outside of platelets, while L-PRP and L-PRF contain leukocytes and various populations of circulating cells [99].

This classification is interesting to correlate with Figure 1, as it highlights the fact that the products from the L-PRF family present all the schematic requirements for bone regeneration from a tissue engineering perspective: cells (leukocytes and many other cell populations) [99], scaffold (fibrin blood clot, often used in bone tissue engineering experiments) [100], and bioactive molecules (growth factors and all the molecules available in platelets, plasma, and leukocytes for starting) [101]. L-PRF has also this particularity to integrate very naturally all these elements, leading to the slow release of growth factors from the L-PRF fibrin matrix and the production of growth factors from the cells living in the gel [98, 101, 102]. For these reasons, L-PRF was often described as an optimized natural blood clot [99], and, as it is often said in orthopedics, there is no good bone healing without adequate bleeding.

Specifically, within the context of this review, it is important to highlight the fact that L-PRF was tested with oral bone mesenchymal stem cells in vitro [103] and revealed itself as a dose-dependent stimulator of proliferation and differentiation of these cells. This result was described also in other studies with osteoblasts [100]. It was considered as the consequence of the coculture between the bone cells and the leukocytes of the L-PRF [100, 103], in the presence of the fibrin matrix and growth factors of the L-PRF clots [98], resulting in complex but natural interactions promoting bone regeneration. L-PRF is therefore in itself an interesting model fulfilling the requirements for bone regeneration presented in Figure 1, and it probably explains that it has been demonstrated to be successful in the treatment of bone defects [94, 95, 104–106], for example, as sole grating material in maxillary sinus floor elevation [107–109]. As an interesting perspective of combined therapy, it seems that one promising novel supportive strategy for the use of MSCs in bone regeneration may be as simple as an optimized L-PRF natural blood clot.

## 4. Conclusion

Bone regeneration based on tissue engineering approaches has a solid background for clinical application in human bone defects. The cell-based, scaffold, bioactive molecule delivery and gene-therapy methods interface and complement each other. However, some of these therapies are still at the preclinical level.

As presented in this paper, many different approaches and biologic agents are being studied. The major challenge for all of them is the timely and sequential organization of events that need to occur in the healing area. The aim is to promote the adequate processes at the precise moment without compromising the normal cell function and overall process. External “on demand” activation technologies are being developed. Additionally, the need for custom medical devices that can be adapted for the patient and the bone defect specific clinical needs will increase the use of 3D printing in the coming years. The association of these techniques with cell-based, bioactive molecules and gene-therapy approaches is a promising and exciting area of research.

However, the current published literature on the clinical application of stem cells for craniofacial bone regeneration is abundant but highly diverse, which reflects (1) the fact that these technologies are relatively new and, therefore, it is difficult to standardize findings and clinical applications; and (2) the number of different potential applications to successfully use cell therapy in the clinical practice is high but still needs to be scientifically proven.

## Figures and Tables

**Table 1:** Principal types and uses of cells in oral tissue regeneration.

Cell type	Origin
Bone marrow stromal cells	Autograft
Adipose stromal cells	Autograft
Periodontal ligament cells	Autograft, allograft, xenograft
Periodontal ligament stem cells	Allograft, autograft

**Table 2:** Randomized clinical trials in the use of MSCs for oral bone tissue regeneration.

Reference	Stem cell type	Colection	Subculture	Origin	*N*	Carrier	Defect type	Graft location	Cover	Control	Time for analysis	Analysis	Primary outcomes	Implants	Restoration	Follow-up after restoration	Implant survival rate	Complications
Da Costa et al. [18]	MSC	BMA	No (whole aspirate)	IB	5 + 5	AB	Horizontal	AM	NO	AB	6 m	CT + Hm	Alveolar thickness gain: 4.6 ± 1.43 versus 2.15 ± 0.47 mm (test versus control); vital bone: 60.7 ± 16.18 versus 41.4 ± 12.5% (test versus control)	Yes (40)	Yes	N/S	100%	N/S

Gimbel et al. [19]	N/S	BMA	No (whole aspirate)	IB	21 tests + 25 controls	CS	Cleft palate	AM	NO	IB	1 d, 1 w, 3 w, 6 w, 6 m	Comfort and complications for donor site	Best results in test group followed by conventional iliac graft	No	No	N/A	N/A	Test: 2 granulation tissues; control: 1 oronasal fistula

Gonshor et al. [15]	MSC	CBA	No	N/S	18: 8 bilats + 10 unilats (=26)	CBA	Sinus lift	PM	NO	Allograft	3.6 ± 0.6 m	H + Hm + CT	Vital bone: 32.5 ± 6.8% (test) - 18.3 ± 10.6% (control)	Yes	No	N/S	N/S	2 patients lost

Kaigler et al. [20]	MSC	BMA	Yes (automated Ixmyelocel-T)	IB	12 + 12	CS	Alveolar reconstruction	M and Mn	CM	CS + CM	6 or 12 w	RX + *μ*CT + H	Linear bone height: 55.3%–78.9 (6 w, control versus test); 74.6%–80.1% (12 w, control versus test)	Yes	Yes	1 year	N/S	N/S

Pelegrine et al. [21]	MSC	BMA	No (whole aspirate)	IB	15 + 15	No	Alveolar reconstruction	AM	NO	No graft	6 m	Clinical data + H + Hm	Horizontal bone loss: 1.14 ± 0.87 versus 2.46 ± 0.4 mm (test versus control); vertical bone loss: 1.17 ± 0.26 mm versus 0.62 ± 0.51 mm (test versus control); new vital bone: 45.47±7.21 versus 42.87 ± 11.33%	Yes (20)	Yes	N/S	100%	5 control sites required regraft at implant placement

Rickert et al. [16]	MNC	BMA	No (BMAC)	IB	12 split mouths (24 sinuses)	BBM	Sinus lift	PM	CM	BBM + retromolar autogenous graft	14.8 ± 0.7 w	Hm	New bone (test versus control): 17.7 ± 7.3% versus 12.0 ± 6.6%	Yes (66 nonsubmerged)	Yes	N/S	N/S	3 implant failures

Sauerbier et al. [22]	MSC	BMA	No (BMAC)	IB	7 patients (12 sites; test) + 4 (6; control)	BBM	Sinus lift	PM	CM	FICOLL	3 m	H + Hm	Similar results for all parameters	Yes	Yes	1 y	98%	1 implant lost in the test group

Sauerbier et al. [17]	MSC	BMA	No (BMAC)	IB	26 patients (45 sinuses) 34 tests/11 controls	BBM	Sinus lift	PM	CM	BBM + Retromolar Autogenous graft	3.46 ± 0.43 m test/3.34 ± 0.42 m control	CT + H + Hm	Radiographic volume gain: 1.74 ± 0.69 versus 1.33 ± 0.62 mL (test versus control); new bone formation: 12.6 ± 1.7 versus 14.3 ± 1.8%	No	No	N/S	N/A	1 inferior alveolar nerve injury during autogenous graft harvesting

Wojtowicz et al. [23]	MNC	BMA	Nonprocessed BMA, CD34+ cells isolated from BMA or PRP	IB	17 (9 CD34+/4 BMA/4 PRP)	BBM	Cystectomy	AMn	FM + CM	No graft	1 & 3 m	RX	Similar trabeculae to nonregenerated bone in BMA and CD34+ groups	No	No	N/S	N/A	N/S

MSC = mesenchymal stem cells; MNC = mononuclear cells; ASC = adipose stem cells; N/S = not specified; BMA = bone marrow aspirate; CBA = cellular bone allograft; BMAC = bone marrow aspirate concentrate; IB = iliac bone; AB = allogenic block; CS = collagen sponge; BBM = bovine bone marrow; AM = anterior maxilla; PM = posterior maxilla; M = maxilla; Mn = mandible; AMn = anterior mandible; CM = collagen membrane; FM = fibrin membrane; d = days; w = weeks; m = months; y = years; H = histology; Hm = histomorphometry; CT = computed tomography; RX = radiography; N/A = not applicable.

**Table 3:** Case series/report in the use of MSCs for oral bone tissue regeneration.

Reference	Study design	Stem cell type	Collection	Subculture	Origin	*N*	Carrier	Defect type	Graft location	Cover	Time for analysis	Analysis	Primary outcomes	Implants	Restoration	Follow-up after restoration	Implant survival rate	Complications
Behnia et al. [24]	CS	MSC	BMA	Yes (2 w, manual, no induction)	IB	2	DBM + calcium sulfate	Cleft palate	AM	NO	4 m	CT	Oronasal fistula closure; 25.6–34.5% bone defect fill	No	No	N/A	N/A	N/S

Behnia et al. [25]	CS	MSC	BMA	Yes (2 w, manual, no induction)	IB	4	HA/TCP + PDGF	Cleft palate	AM	FC	3 m	CT	Oronasal fistula closure; 51.3% bone defect fill	No	No	N/A	N/A	N/S

Cerruti et al. [26]	CS	MNC	BMA	No (whole aspirate)	IB and SB	32	AB + PPP + PRP	Vertical, horizontal, sinus lift	AM and PM	N/S	4 m	H + CT	Width: 6–14 mm (AM); height: ≈10 mm (AM) and 6 -> 15 mm (PM)	Yes	Yes	4 years	100%	1 graft not integrated; 1 sinus infection

Hernández-Alfaro et al. [27]	CR	MSC	BMA	No (BMAC)	IB	1	DBB + BMP-2	Ameloblastoma resection	PMn	CM	9 m	CT + H	Adequate bone formation	Yes	Yes	1 year	100%	N/S

Hibi et al. [28]	CR	MSC	BMA	Yes (4 w, manual, osteogenic induction with 100 nM dexamethasone, 10 mM b-glycerophosphate, and 50 mg/mL ascorbic acid-2- phosphate)	IB	1	PRP	Cleft palate	AM	TM	3–6–9 m	CT	79.1% bone coverage	No	No	N/S	N/A	N/S

Lee et al. [29]	CR	MSC	BMA	Yes (4 w, manual, osteogenic induction by 50 *μ*g/mL of L-ascorbic acid, 10-mmol/L glycerol phosphate, and 10*E* − 7-mol/L dexamethasone)	IB	1	FDAB + Fibrin	Hemangioma resection	PMn	TM	12 m	CT + H	New bone formation, graft contains live osteocytes, enough bone height for implant placement	Yes	No	N/S	N/S	N/S

Meijer et al. [30]	CR	MSC	BMA	Yes (manual, osteogenic induction by dexamethasone)	IB	6	HA	Sinus lift and other defects	PM and PMn	NO	4 m biopsy/3, 6, 9, 15 m RX	RX + Clinical data + Hm	Adequate bone mainly induced by the carrier/adequate radiographic bone reconstruction	Yes	Yes	15 m	N/S	1 implant failure

Sándor et al. [31]	CR	ASC	SAT	Yes (3 w, manual, no induction)	AAW	1	B-TCP + BMP-2	Ameloblastoma resection	AMn	NO	10 m	Panoramic RX + Hm	Successful bone reconstruction, implant placement, and prosthetic rehabilitation	Yes	Yes	N/S	N/S	N/S

Sándor et al. [32]	CS	ASC	SAT	Yes (3 w, manual, no induction)	AAW	3	B-TCP + BMP-2	Ameloblastoma resection	Mn	TM	1w, 1–12 m	Clinical data + RX	Successful bone reconstruction, uneventful healing	2 patients (7 implants)	Yes	27–51 m	86%	N/S

Sauerbier et al. [33]	CR	MSC	BMA	No (BMAC)	IB	2 patients	BBM	Vertical, horizontal	PM	CM	7 m or 4 m	H + RX	51.6% and 20.0% new bone formation, respectively	Yes	Yes	2 y	100%	NO

Schmelzeisen et al. [34]	CR	N/S	BMA	No (BMAC)	IB	1 (2 sinuses)	BBM	Sinus lift	PM	N/S	3 m	Hm	29.1% BBM; 26.9% NBF	No	No	N/S	N/A	N/S

Shayesteh et al. [35]	CR	MSC	BMA	Yes (4 w, manual, no osteogenic induction)	IB	7	HA/TCP	Sinus lift	PM	CM	3 m, 1 y	RX + Hm	New bone: 41.34%; radiographic bone height: 2.25–12.08–10.83 (baseline-postgraft-1 y)	Yes (30)	Yes	6 m	93%	2 implants lost before restoration

Smiler et al. [36]	CS	N/S	BMA	No (whole aspirate)	IB	5 patients (7 sites)	Xenograft, allograft, or alloplastic graft (*β*-TCP)	Sinus lift or horizontal	PM	CM + TM	4–7 m	H + Hm	23–45% of new bone formation, no differences between carriers are statistically reported	No	No	N/S	N/A	N/S

Soltan et al. [37]	CS	N/S	BMA	No (whole aspirate)	IB	5	AB	Sinus lift or horizontal	AM and PM	N/S	8–12 m	H + Hm	89% new vital bone (54% bone, 46% marrow)	Yes	Yes	N/S	N/S	N/S

Soltan et al. [38]	CS	N/S	BMA	No (whole aspirate)	IB	2 patients/6 sites	HA or particulate allograft	Horizontal	PM and PMn	N/S	4–6 m	H + Hm	34–45% new bone, no statistical differences reported	Yes	Yes	N/S	N/S	N/S

Ueda et al. [39]	CS	MSC	BMA	Yes (4 w, manual, osteogenic induction by dexamethasone, sodium *β*-glycerophosphate, and L-ascorbic acid 2-phosphate)	IB	6	*β*-TCP + PRP	Sinus lift	PM	TM	6 m	Clinical data + CT	7.3 ± 4.6 mm height gain	Yes (20)	Yes	12 m	100%	2 sinus membranes perforation, with minor nasal bleeding

Ueda et al. [40]	CS	MSC	BMA	Yes (4 w, manual, osteogenic induction by dexamethasone, sodium *β*-glycerophosphate, and L-ascorbic acid 2-phosphate)	IB	14 (6 sinus lifts/8 onlay graftings)	PRP	Sinus lift or vertical	PM	Titanium reinforced CM for vertical ridge augmentation	4.8 m	Clinical data + RX	8.7 mm height gain in sinus; 5 mm in ridges	Yes	Yes	2–5 y	100%	4 sinus membranes perforation

Wongchuensoontorn et al. [41]	CR	MSC	BMA	No (BMAC)	IB	1	IB	Mn fracture	PMn	CM	4 m	Panoramic RX	Mandibular fracture consolidation	No	No	4 m	N/A	N/S

Yamada et al. [42]	CR	MSC	BMA	Yes (4 w, manual, osteogenic induction by 100 nM dexamethasone, 10 mM sodium *β*-glycerophosphate, and 25 mg/mL L-ascorbic acid 2-phosphate)	IB	1	PRP	Vertical, horizontal	PMn	CM + TM	7 m	CT + H	4.2 mm bone height gain, new mature bone formation	Yes (3)	Yes	2 y	100%	N/S

CS = case series; CR = case report; MSC = mesenchymal stem cells; MNC = mononuclear cells; ASC = adipose stem cells; N/S = not specified; BMA = bone marrow aspirate; SAT = subcutaneous adipose tissue; BMAC = bone marrow aspirate concentrate; IB = iliac bone; SB = sternum bone; AAW = anterior abdominal wall; DBM = demineralized bone marrow; HA/TCP = hidroxyapatite/tricalcium phosphate; PDGF = platelet derived growth factor; PPP = platelet-poor plasma; PRP = platelet-rich plasma; AB = allograft block; DBB = demineralized bovine bone; BBM = bovine bone marrow; IB = iliac bone; AM = anterior maxilla; PM = posterior maxilla; AMn = anterior mandible; PMn = posterior mandible; M = maxilla; Mn = mandible; CM = collagen membrane; FM = fibrin membrane; FC = fibrin clot; TM = titanium mesh; d = days; w = weeks; m = months; y = years; H = histology; Hm = histomorphometry; CT = computed tomography; RX = radiography; N/A = not applicable.
